# Association between body composition and blood pressure in normal-weight Chinese children and adolescents

**DOI:** 10.1186/s12887-022-03289-z

**Published:** 2022-05-02

**Authors:** Ling Bai, Jinyu Zhou, Lingling Tong, Wenqing Ding

**Affiliations:** 1 School of Public Health and Management, Ningxia Medical University, Shengli Street, Xingqing District, Yinchuan, 750004 Ningxia China; 2 Key Laboratory of Environmental Factors and Chronic Disease Control, No.1160, Shengli Street, Xingqing District, Yinchuan, 750004 Ningxia China

**Keywords:** Normal weight, Fat mass percentage, Visceral fat level, Blood pressure, Body composition

## Abstract

**Background:**

The aim of this study was to assess the associations of body fat distribution and lean body mass (LBM) with blood pressure (BP) in normal-weight Chinese children and adolescents.

**Methods:**

A total of 898 normal-weight Chinese children and adolescents, aged 10–18 years, were included this cross-sectional study via a cluster sampling method. The bioelectrical impedance analysis (BIA) was used to measure body composition. The participants were measured for blood pressure (BP) using a calibrated electronic sphygmomanometer according to the standard method by the "American Hypertension Education Project Working Group".

**Results:**

Body composition was related to abnormal BP in normal-weight children and adolescents. After the model adjusted for age, smoking, and drinking, regression analysis showed that fat mass percentage (FMP) was negatively associated with abnormal BP, while LBM was positively associated with abnormal BP in boys(*P* < 0.05). Whereas FMP and visceral fat level (VFL) were positively associated with abnormal BP in girls (*P* < 0.05).

**Conclusions:**

There are sex differences in the relationships between total body fat, visceral fat and lean body mass with abnormal BP in normal-weight youths. Therefore, it is of great significance to pay attention to the relative influence of the body composition of the boys and girls in the prevention and treatment of hypertension in youths.

## Background

The prevalence of hypertension in children and adolescents is increasing worldwide over time. A meta-analysis of 301 articles showed that elevated BP in childhood early could lead to hypertension in adulthood, which is considered to be a main risk factor for the worldwide burden of cardiovascular diseases (CVDs) [1], and it has caused millions of deaths and disability-adjusted life years in recent years [2]. Notably, some studies with large sample sizes (e.g., 58,899 adolescents) showed that prevalence of elevated blood pressure (BP) and high BP are also increasing in Chinese children and adolescents [3–5]. Elevated BP was defined as SBP/DBP ≥ 90th (or ≥ 120/80 mm Hg) and < 95th percentile by sex, age, and height; high BP was defined as SBP/DBP ≥ 95th percentile by sex, age, and height [6].

Higher body mass index (BMI) has been illustrated to be strongly associated with elevated BP and high BP [7]. Another cross-sectional study of a large sample of 58,899 adolescents from China, America and five other countries also showed that a normal range BMI was associated with an increased risk of elevated BP [3]. The reasons for this are uncertain, and they may be related to the limitations of BMI itself. BMI is a general indicator for assessing overall obesity, but it cannot distinguish between body fat distribution and lean body mass (LBM). This is important because different fat distribution indicators have different effects on BP [8]. Therefore, it may help explain why normal weight adolescents are still at health risk after investigating the body fat distribution or LBM has a greater impact on BP in children and adolescents with normal BMI.

There are two different but representative indicators of body fat distribution: fat mass percentage (FMP) and visceral fat level (VFL, indicating that visceral adipose tissue includes intra-abdominal, perirenal and pericardial adipose tissue) [9], which reflect the proportion of total body fat and visceral fat respectively, and are linked to abnormal BP [10–12]. Furthermore, not only is excess fat associated with abnormal BP, but LBM is also connected [13]. However, the contribution of these risk indicators to the development of abnormal BP in normal-weight children and adolescents is unknown. Abnormal BP was defined as SBP or DBP ≥ 90th percentile by age, sex and height according to the Chinese reference standard for BP in children and adolescents [14].

Therefore, the aim of this study was to assess the associations of body composition with abnormal BP in normal-weight children and adolescents, and differences in body composition between adolescent boys and girls.

## Methods

### Study participants

A cross-sectional study was designed to collect data from 1175 children and adolescents aged 10–18 years, who were selected from three junior schools and two high schools in China between 2017 and 2019 by cluster sampling. Schools were first chosen by a convenient sampling method, then grades, classes were randomly selected from each grade in the survey (a total of 11 whole classes from the three junior high schools and 29 from the three high schools) The sample size was calculated based on the following formula: $n=\frac{{Z}_{\alpha }^{2}\times pq}{{d}^{2}}$(α is the significance level, p is the prevalence of elevated BP [15], d is the tolerance error and Z is the significance test statistic; α = 0.05, *p* = 16.3%, d = 0.15p, q = 1-p), assuming a non-response rate of 15% and a final required sample size of approximately *n* = 1009, this study ultimately surveyed 1175 children and adolescents. All study subjects participated in questionnaires, anthropometric measurements and body composition examinations. Of the 1175 participants who participated in this study, a total of 898 normal-weight (52.2% boys) samples were remained in the final analysis after excluding 277 participants with overweight (*n* = 216) and obesity (*n* = 61). The study was conducted according to the standards of the Declaration of Helsinki and approved by the Ethics Committee of Ningxia Medical University (2021-G053), all methods were performed in accordance with the relevant guidelines and regulations. Informed consent was signed by all participants and their parents/ guardians.

### General information

Data for the study were collected by trained staff at each selected school according to the measurement criteria of each instrument. The questionnaire was self-filled and consisted mainly of demographic characteristics (sex, age, date of birth, smoking and drinking, etc.). It should be noted the classification of smoking were: 0. Never smoked; 1. Try to smoke (try to smoke, even if it's just one or two); 2. Recently smoked (have smoked at least 1 cigarette in the past 30 days), and the classification of drinking are:0. Never drink alcohol; 1. Try to drink alcohol (have drunk at least half a bottle or a can of beer, a small cup of liquor, etc.); 2. Drinking alcohol now (in the past 30 days, at least a glass of alcohol has been drunk one day). Height and weight were measured using a mechanical stadiometer (Model: ZH7082) and an electronic scale (Model: RGT-140). Both were measured twice and averaged for inclusion in the final analysis, to an accuracy of 0.1 cm and 0.1 kg for height and weight respectively. The height, weight and waist circumference were measured according to the requirements of the "2014 National Student Physical Fitness and Health Survey Manual" [16]. Height measurement: the subjects were barefoot, the torso was naturally straight, and the eyes were straight ahead. The upper limbs drooped naturally and the legs were straightened. Bring the heels together, and the toes were separated by about 45 degrees. The heel and sacrum and between the shoulder blades were in contact with the upright post, forming a "three points and one line" standing posture. Weight measurement: the subjects stood barefoot and naturally stood in the center of the weight scale pedal to keep the body stable. When measuring the waist circumference (WC), the subjects stood naturally with their feet 30–40 cm apart, and place a nylon tape measured between the upper hip bone and the lower thoracic cavity (usually the natural narrowest part of the waist), at the end of normal expiration, around the abdomen in a horizontal direction twice, took the average to an accuracy of 0.1 cm. BMI was calculated as weight divided by the squared of height (kg/m^2^). Normal weight was defined as the reference standard for overweight and obesity in children and adolescents as recommended by the International Obesity Task Force (IOTF): BMI ≤ international cut off points for BMI for overweight and obesity by age and sex [17].

### BP Measurements and body fat composition examinations

Blood pressure (BP) was measured using a calibrated electronic sphygmomanometer (Model: OMRON HEM-7012, Omron Healthcare, Kyoto, Japan) according to the standard method by the "American Hypertension Education Project Working Group" [18]. A suitable cuff was chosen for the measurement (7 cm, 9 cm, 12 cm, etc. for BP measurement in children and adolescents) and the subject was seated facing the measurer and BP was measured on the right upper arm with the elbow at the same level as the sphygmomanometer and the heart. Systolic BP (SBP) and diastolic BP (DBP) were measured three times at 1-min intervals, and the average of the last two readings was recorded for the final analysis (a third measurement was taken if the difference between the first two blood pressure values exceeded 10 mm Hg (1 mm Hg = 0.133 kPa)).

The participants were assessed for body composition using bioelectrical impedance analyzer (Model: InBody-370, Biospace of Korea, Seoul, Korea). Under the condition of fasting condition, the test subject stood barefoot on the instrument, stepped on the designated electrodes on both sides of the forefoot and heel respectively, held the handle and relaxed the whole body, waited for the instrument to automatically complete the test, and completed the calculation based on the measured resistance rate and generate a test report. In this study, body fat mass (BFM), fat mass percentage (FMP, calculated as BFM divided by total body mass), visceral fat level (VFL), VFL/FMP ratio (indicating the degree of visceral fat deposition in the body) and lean body mass (LBM) were included in the final analysis.

### Statistical analysis

In this study, statistical analyses were accomplished by SPSS 26.0. Sex-stratified analyses were conducted. Continuous variables were described by mean (standard deviation), discrete variables by median (25th percentile,75th percentile), and categorical variables were calculated by frequencies and percentages. For normal variables, sex differences were tested by t-test and differences between quartiles by ANOVA; For skewed variables, differences between groups were tested by Mann–Whitney U-test, and categorical variables with χ^2^ test. A binary logistic regression analysis was performed with FMP, VFL, VFL/FMP ratio and LBM as independent variables and abnormal BP as the dependent variable. Model 1 was a separate regression model for various body composition indexes and abnormal BP, and Model 2 to Model 5 were regression models with different body composition variables included simultaneously in the corresponding equations, respectively. All models were adjusted for age, smoking and drinking (with an additional adjustment for sex in the total population). The OR values and their 95% CIs for abnormal BP risk per 1-SD increase in the above indicators were estimated. *P* < 0.05 was regarded as statistically significant.

## Results

The main characteristics of the study subjects are shown in Table 1. Overall, a total of 898 (52.2% boys) normal-weight children and adolescents were included in the final analysis, of whom 19.0% were detected with abnormal BP (45.6% boys). Boys had higher age, weight, height, SBP and LBM than girls, but lower BMI, WC, BFM, FMP, VFL and VFL/FMP ratio respectively (*P* < 0.05), and differences in smoking and drinking between the boys and girls (*P* < 0.05).

**Table 1:** Characteristics of the study participants

Characteristics	Total	Boys	Girls	*p-*value
N (%)	898(100)	469(52.2)	429(47.8)	
Age, years	15.1(2.1)	15.3(2.0)	14.9(2.2)	0.004
Weight, kg	53.5(8.5)	56.0(9.3)	50.8(6.5)	< 0.001
Height, cm	167.1(8.8)	171.2(9.0)	162.5(5.8)	< 0.001
BMI, kg/m^2^	19.1(2.0)	19.0(2.0)	19.2(2.0)	0.019
WC, cm	70.9(5.9)	70.3(6.0)	71.6(5.7)	0.001
Blood pressure				
SBP, mm Hg	111.4(10.0)	113.9(10.3)	108.5(8.8)	< 0.001
DBP, mm Hg	68.1(7.3)	67.9(7.4)	68.3(7.1)	0.460
Abnormal BP n (%)	171(19.0)	78(45.6)	93(54.4)	0.054
Body fat variables				
BFM, kg^a^	10.1(6.8, 13.7)	7.4(5.5, 10.2)	12.8(10.3, 16.3)	< 0.001
FMP, %^a^	19.2(13.0, 25.7)	13.5(10.6, 17.3)	25.6(21.4, 29.5)	< 0.001
VFL^a^	4.0(2.0, 5.0)	2.0(2.0, 4.0)	5.0(4.0, 6.0)	< 0.001
VFL/FMP^a^ ratio, %^a^	19.2(13.0, 22.3)	18.2(14.7, 22.4)	19.6(17.5, 22.3)	< 0.001
LBM, kg^a^	41.3(36.9,48.8)	48.5(43.3,53.1)	37.6(35.1,40.2)	< 0.001
Smoking, n (%)				
Never	624(78.3)	259(64.0)	365(93.1)	< 0.001
Try	124(15.6)	100(24.7)	24(6.1)	
Current	49(6.1)	46(11.4)	3(0.8)	
Drinking, n (%)				
Never	481(60.4)	205(50.9)	276(70.2)	< 0.001
Try	221(27.8)	134(33.3)	87(22.1)	
Current	94(11.8)	64(15.9)	30(7.6)	

Characteristics are shown as mean (SD), median (25th, 75th percentile) or n (%)

^a^Skewed distribution; Abnormal BP, SBP/DBP ≥ *P*_90_ by sex, age and height; *BFM* Body fat mass, *FMP* Fat mass percentage, *VFL* Visceral fat level, VFL/FMP ratio, which reflects the degree of visceral fat deposited, *LBM* Lean body mass; *P*-value denote result of t-test, *χ*^*2*^ test or Mann–Whitney non-parametric test between boys and girls

The relationships between mean BP z-scores and different quartiles of body composition variables were found in both boys and girls (Table 2). Boys with a higher FMP instead had lower SBP z-scores compared to boys with a lower FMP(*P* < 0.05). The difference in SBP z-scores and DBP z-scores between the different VFL/FMP quartiles in the total population and girls (except for DBP z-scores) were statistically significant, with higher VFL/FMP groups also having higher SBP z-scores(*P* < 0.05). In addition, Table 2 shows the increasing trend in SBP z-scores with the quartiles of LBM in both genders (*P* < 0.05).

**Table 2:** Mean blood pressure z-scores across quartile of body fat distribution variables by sex

		Q1	Q2	Q3	Q4	*P* value
***Boys***						
FMP		10.40(8.70,11.80)	15.50(14.20,17.20)	22.10(20.60,23.80)	29.10(27.85,32.50)	
	SBP Z-score	0.10(0.99)	0.00(1.01)	-0.17(0.93)	-0.88(0.42)	**0.002**
	DBP Z-score	-0.02(0.98)	0.09(1.04)	-0.07(0.99)	-0.43(0.49)	0.249
VFL		2.0(1.0,2.0)	3.0(3.0,4.0)	5.0(5.0,5.0)	7.0(6.0,9.0)	
	SBP Z-score	0.05(0.98)	-0.05(1.03)	0.14(0.95)	-0.42(0.83)	0.104
	DBP Z-score	-0.01(1.00)	-0.03(0.96)	0.25(1.07)	-0.18(0.90)	0.305
VFL/FMP		13.29(10.75,14.93)	17.54(16.81,18.18)	20.83(20.07,21.52)	25.06(23.55,27.16)	
	SBP Z-score	-0.12(1.00)	-0.01(0.93)	0.02(1.10)	0.15(0.92)	0.162
	DBP Z-score	-0.08(1.01)	-0.06(0.94)	-0.03(1.03)	0.19(0.95)	0.124
LBM		32.30(29.55,34.15)	39.30(38.15,40.35)	45.75(43.70.47.50)	53.40(51.10,56.30)	
	SBP Z-score	-0.54(0.68)	-0.16(0.95)	-0.03(1.11)	0.16(0.92)	** < 0.001**
	DBP Z-score	-0.16(0.80)	-0.07(1.02)	-0.02(1.13)	0.06(0.91)	0.525
***Girls***						
FMP		12.30(11.75,12.60)	17.05(15.75,18.25)	22.85(21.30,24.60)	29.80(27.80,32.10)	
	SBP Z-score	-0.30(1.48)	0.02(0.76)	0.00(0.98)	0.00(1.04)	0.886
	DBP Z-score	-0.40(1.15)	-0.12(0.88)	-0.07(0.96)	0.11(1.03)	0.173
VFL		2.0(2.0,2.0)	4.0(3.0,4.0)	5.0(5.0,5.0)	7.0(6.0,8.0)	
	SBP Z-score	-0.27(1.02)	-0.03(0.91)	-0.07(1.03)	0.10(1.03)	0.299
	DBP Z-score	-0.41(1.05)	-0.07(0.93)	-0.04(1.01)	0.15(1.02)	0.055
VFL/FMP		15.21(14.25,15.92)	18.08(17.15,18.62)	20.44(19.92,21.54)	24.56(23.35,26.32)	
	SBP Z-score	-0.01(1.00)	-0.20(0.97)	0.13(0.99)	0.08(0.98)	**0.048**
	DBP Z-score	-0.06(1.03)	-0.14(0.98)	0.08(0.98)	0.09(0.98)	0.216
LBM		34.80(32.90,35.90)	38.75(37.80,40.00)	43.60(42.40,44.88)	50.20(48.90,50.75)	
	SBP Z-score	-0.11(0.99)	-0.03(0.98)	0.40(0.87)	-0.11(2.03)	**0.004**
	DBP Z-score	0.01(1.00)	-0.09(0.95)	0.19(1.07)	0.25(0.84)	0.240
***Total***						
FMP		10.5(8.7,11.9)	15.9(14.4,17.5)	22.7(21.1,24.5)	29.8(27.8,32.1)	
	SBP Z-score	0.09(1.00)	0.00(0.95)	-0.04(0.96)	-0.06(1.04)	0.395
	DBP Z-score	-0.03(0.98)	0.03(1.00)	-0.07(0.97)	0.07(1.01)	0.467
VFL		2.0(1.0,2.0)	4.0(3.0,4.0)	5.0(5.0,5.0)	7.0(6.0,8.0)	
	SBP Z-score	0.02(0.98)	-0.04(0.97)	-0.00(1.00)	0.03(1.02)	0.871
	DBP Z-score	-0.04(1.01)	-0.05(0.94)	0.05(1.04)	0.10(1.00)	0.335
VFL/FMP		14.0(11.5,15.4)	17.8(17.0,18.5)	20.7(20.0,21.5)	24.9(23.5,26.6)	
	SBP Z-score	-0.09(1.00)	-0.12(0.96)	0.08(1.04)	0.12(0.95)	**0.002**
	DBP Z-score	-0.08(1.02)	-0.11(0.97)	0.04(1.00)	0.14(0.96)	**0.034**
LBM		34.35(32.50,35.80)	38.90(37.90,40.10)	44.85(43.30,47.00)	53.40(51.00,56.30)	
	SBP Z-score	-0.20(0.95)	-0.06(0.98)	0.10(1.06)	0.16(0.94)	**0.001**
	DBP Z-score	-0.02(0.97)	-0.09(0.96)	0.04(1.11)	0.06(0.90)	0.365

Data are shown as mean (SD); *P*-values indicate differences in mean BP values across quartiles of body fat distribution variables by ANOVA

Table 3 presented the odd ratios (OR) with 95%CI for body composition variables with abnormal BP. When variables were separately included in the regression model, LBM in boys, FMP and VFL in girls were positively associated with abnormal BP, respectively (Model 1, *P* < 0.05). FMP was negatively associated with abnormal BP in boys when both FMP and VFL or VFL/FMP ratios were included in the same adjusted models for confounding factors (Model 2 and model 3, *P* < 0.05). In contrast, when both LBM and FMP or VFL included in the same adjusted models for confounding factors, LBM was positively associated with abnormal BP in boys, whereas FMP and VFL were positively associated with abnormal BP in girls (Models 4 and model 5, *P* < 0.05).

**Table 3:** The odd ratios (OR) with 95%CI for body composition variables with abnormal BP

variables	Total	boys	girls
***Model1***			
FMP	1.02(0.98-1.05)	0.97(0.92-1.02)	1.06(1.02-1.11)^**^
VFL	1.12(1.02-1.22)^*^	1.01(0.88-1.17)	1.22(1.07-1.39)^**^
VFL/FMP ratio	1.02(1.00-1.03)	1.01(0.99-1.03)	1.10(1.03-1.17)^*^
LBM	0.99(0.96-1.02)	1.05(1.01-1.09)^*^	1.03(0.97-1.10)
***Model2***			
FMP	0.96(0.92-1.01)	0.94(0.88-1.00)^*^	1.00(0.91-1.10)
VFL	1.22(1.05-1.42)^*^	1.16(0.96-1.40)	1.22(0.94-1.58)
***Model3***			
FMP	0.91(0.84-0.99)^*^	0.84(0.73-0.97)^*^	1.12(0.89-1.39)
VFL	1.52(1.11-2.08)^**^	1.89(1.07-3.33)^*^	0.71(0.25-2.00)
VFL/FMP ratio	0.97(0.93-1.01)	0.95(0.90-1.01)	1.17(0.88-1.56)
***Model4***			
LBM	1.04(1.01-1.08)^*^	1.04(1.00-1.08)^*^	1.03(0.97-1.10)
FMP	1.02(0.99-1.08)	0.98(0.93-1.03)	1.06(1.02-1.12)^**^
***Model5***			
LBM	1.04(1.00-1.07)^*^	1.05(1.01-1.09)^*^	1.01(0.95-1.08)
VFL	1.11(1.02-1.22)^*^	1.01(0.87-1.17)	1.22(1.07-1.39)^**^

*P<0.05, **P<0.01. All models were adjusted for age, smoking and drinking (with an additional adjustment for sex in the total population)

## Discussion

Based on the data from 898 children and adolescents of different middle and high schools, we examined the association between body composition and abnormal BP among normal-weight children and adolescents. The results show that after adjusting for confounding factors, when both FMP and VFL or VFL/FMP ratio were included, FMP was negatively associated with abnormal BP in normal-weight boys; whereas when both LBM and FMP or VFL were included, LBM was positively associated with abnormal BP in normal-weight boys, but FMP and VFL were positively associated with abnormal BP in normal-weight girls, respectively. These results suggest that body fat percentage, visceral fat and lean body mass play different roles in abnormal blood pressure for normal-weight boys and girls, respectively.

A study of 977 normal-weight participants (19.7% men) from Canada showed that the elevated FMP is at augmented risk of metabolic abnormality including hypertension, and results remained significant after further adjustment for WC [19]. Another study found that high BMI, but not WC and FMP, was related to high risk of hypertension in normal-weight youths [20]. Whereas we found that FMP was negatively associated with abnormal blood pressure in boys after adjusting for confounding factors, which is inconsistent with the findings above. However, pausova et al. also found that BP was less strongly and negatively associated with total body fat in boys [21], which is similar to the results of present study. It means that FMP may be a protective factor for BP in normal-weight boys. In this study, the protective mechanism of FMP on blood pressure in boys may be related to subcutaneous fat: after controlling their visceral fat with normal weight, the subcutaneous fat in adiposity played an important role in protecting blood pressure. Bidulescu et al. found that subcutaneous adipose tissue of the abdominal appeared as a protective fat depot in male [22]. A study from China suggested that after adjustment for confounding factors, leg fat mass to total fat mass was negatively associated with BP in the non-obese subjects [23]. Leg fat is consisted only of subcutaneous fat without visceral fat. Besides, some other studies also found that ﻿higher leg fat mass is protective against cardiovascular risk factors and their clustered risk in children and adolescents [24, 25]. This may partly explain that the FMP of boys has a protective effect on BP. But more research is needed to further elucidate the mechanisms underlying subcutaneous fat against blood pressure and even cardiovascular risk factors. At the same time, we found that LBM was positively associated with abnormal blood pressure in normal-weight boys. A previous study showed that greater lean body mass index (LBMI) was related to greater odds of high BP, independent of FMI [26]. Another study also found that LBMI was a better and more important predictor of BP than BMI in British children, and that the use of LBMI rather than BMI should be considered when considering the effect of weight status on BP [12]. These findings above were similar to our result.

Notably, FMP and VFL were positively associated with abnormal blood pressure among girls respectively, which were different from boys. This is similar to the findings of Malden et al. and others [27, 28]. Other studies also suggested that fat mass and lean mass may have a similar quantitative effect on BP in healthy-weight girls [29–31]. However, we found that the association between LBM and abnormal blood pressure in girls was not significant after adjusting for confounding factors, which is inconsistent with the results of some studies. Some scholars have found that visceral fat and LBM were positively associated with BP in both boys and girls [27, 32, 33]. And another study found that a high percentage of lean body mass was related to a low BP and low hypertension risk among healthy Chinese children [34]. On the one hand, these inconsistencies may be related to different populations, ethnicities, and regions as well as instruments for measuring body composition. But on the other hand, they may reflect the fact that differences between boys and girls in adolescence may lead to different risks of occurrence for abnormal BP through different distribution of body composition.

Regarding the sex difference in body composition, the different health risks may be caused by the following reasons. Firstly, males have greater lean body mass and mineral mass, a lower fat mass than females, and girls start puberty earlier and suffer a much faster pubertal transition than boys, then they possess relatively greater total adiposity in the same period. This sex difference in body composition are mainly traceable to the function of sex steroid hormones in both boys and girls, which drive the final stature during pubertal development [35]. Secondly, higher LBM is usually associated with a high proportion of Type II and Type IIx muscle fibres, and Type II muscle fibres not only have the greatest capacity for muscle hypertrophy but also are positively associated with the risk of resting BP. Finally, several studies have shown that increased pro-inflammatory activity and lipocalin of visceral fat are associated with risk of abnormal blood pressure and that there are sex differences in each of these factors [36–40]. Abdominal visceral fat had negatively associated with serum adiponectin concentrations in female but not male [22]. Besides, sympathetic activation may be an underlying link between visceral fat and hypertension, and sympathetic activation differs in boys and girls [10]. But the potential mechanisms underlying the association between sex-specific body composition and abnormal blood pressure remain unclear and further research is needed to elucidate them.

The main strength is that the study focuses on normal-weight adolescents and the protective effect of fat mass percentage on BP in boys after adjusting for visceral fat and the remaining confounders. However, several limitations should be noted here. Firstly, the study was based on a cross-sectional design and could not assess the causal relationship between body composition and abnormal blood pressure. More cohort studies are necessary to assess the long-term effects of body composition and abnormal BP in children and adolescents. Secondly, underweight study subjects were not excluded according to the IOTF criteria. Thirdly, the questionnaire lacks questions related to monthly household income and parents' occupations, so it is impossible to assess the family socioeconomic status of the study participants and to include them in our analysis, which may affect our findings. Finally, other regional fats were not involved, which may have overlooked the effect of these regional fats on abnormal BP.

## Conclusion

This cross-sectional study suggests that different body composition is associated with blood pressure in normal-weight Chinese children and adolescents, and their relative contributions varies between boys and girls. Greater attention needs to be paid to the effect of the body composition on blood pressure in both boys and girls when screening for blood pressure abnormalities.

## Data Availability

The datasets used and/or analysed during the current study are available from the corresponding author on reasonable request.

## Abbreviations

CVDs: Cardiovascular diseases
BP: Blood pressure
BMI: Body mass index
BFM: Body fat mass
FMP: Fat mass percentage
LBM: Lean body mass
VFL: Visceral fat level

## References

[1] Chen X, Wang Y (2008). Tracking of blood pressure from childhood to adulthood: a systematic review and meta-regression analysis. Circulation.

[2] Group GNDC (2017). Global, regional, and national burden of neurological disorders during 1990–2015: a systematic analysis for the global burden of disease study 2015. Lancet Neurology.

[3] Wang M, Kelishadi R, Khadilkar A, Mi Hong Y, Nawarycz T, Krzywińska-Wiewiorowska M (2019). Body mass index percentiles and elevated blood pressure among children and adolescents. J Hum Hypertens.

[4] Lu Y, Luo B, Xie J, Zhang X, Zhu H (2018). Prevalence of hypertension and prehypertension and its association with anthropometrics among children: a cross-sectional survey in Tianjin China. J Hum Hypertens.

[5] Hu J, Shen H, Wu J, Xiao Q, Chu G, Teng C (2019). Prevalence of high blood pressure and high normal blood pressure among 7- to 17-year-old children and adolescents in developed regions, China from 2014 to 2017: using new national blood pressure reference for Chinese children and adolescents. J Hum Hypertens.

[6] Xi B, Zong X, Kelishadi R, Hong Y, Khadilkar A, Steffen L (2016). Establishing international blood pressure references among nonoverweight children and adolescents aged 6 to 17 years. Circulation.

[7] Parker E, Sinaiko A, Kharbanda E, Margolis K, Daley M, Trower N (2016). Change in weight status and development of hypertension. Pediatrics.

[8] Katzmarzyk PT, Shen W, Baxter-Jones A, Bell JD, Butte NF, Demerath EW (2012). Adiposity in children and adolescents: correlates and clinical consequences of fat stored in specific body depots. Pediatr Obes.

[9] González-Muniesa P, Mártinez-González M, Hu F, Després J, Matsuzawa Y, Loos R (2017). Obesity. Nat Rev Dis Primers.

[10] Pausova Z, Abrahamowicz M, Mahboubi A, Syme C, Leonard GT, Perron M (2010). Functional variation in the androgen-receptor gene is associated with visceral adiposity and blood pressure in male adolescents. Hypertension.

[11] Matsuzawa Y (2010). Establishment of a concept of visceral fat syndrome and discovery of adiponectin. Proc Jpn Acad Ser B Phys Biol Sci.

[12] DeyhimbASaF (2012). assessing indicators of central obesity as hypertensive risk factors. J Student Res.

[13] Duncan MJ, James L, Griffiths L (2011). The relationship between resting blood pressure, body mass index and lean body mass index in British children. Ann Hum Biol.

[14] Fan H, Yan Y, Mi J (2017). Updating blood pressure references for Chinese children aged 3–17 years. Chinese J Hypert.

[15] Zhai Y, Li W, Shen C, Qian F, Shi X (2015). Prevalence and correlates of elevated blood pressure in Chinese children aged 6–13 years: a nationwide school-based survey. Biomed Environ Sci.

[16] National Student Physical Fitness and Health Survey Group (2014). 2014 National Student Physical Fitness and Health Survey Handbook [M].

[17] Tim J Cole MCB, Katherine M Flegal, William H Dietz. Establishing a standard definition for child overweight and obesity worldwide: international survey. BMJ. 2000;320(7244):1240-3. 10.1136/bmj.320.7244.1240 PMC27365 10797032

[18] Falkner B, Daniels S (2004). Summary of the fourth report on the diagnosis, evaluation, and treatment of high blood pressure in children and adolescents. Hypertension.

[19] Shea JL, King MT, Yi Y, Gulliver W, Sun G (2012). Body fat percentage is associated with cardiometabolic dysregulation in BMI-defined normal weight subjects. Nutr Metab Cardiovasc Dis.

[20] Xu RY, Zhou YQ, Zhang XM, Wan YP, Gao X (2018). Body mass index, waist circumference, body fat mass, and risk of developing hypertension in normal-weight children and adolescents. Nutr Metab Cardiovasc Dis.

[21] Pausova Z, Mahboubi A, Abrahamowicz M, Leonard GT, Perron M, Richer L (2012). Sex differences in the contributions of visceral and total body fat to blood pressure in adolescence. Hypertension.

[22] Bidulescu A, Liu J, Hickson DMA (2013). Gender differences in the association of visceral and subcutaneous adiposity with adiponectin in African Americans: the Jackson heart study. BMC Cardiovasc Disord.

[23] Yan S, Zhao X, Shen X, Yang L, Yuan X, Huang L (2013). Abnormal regional body fat distribution also exists in non-obese subjects with high blood pressure. Clin Exp Hypertens.

[24] Yan Y, Liu J, Zhao X, Cheng H, Huang G, Mi J (2019). Regional adipose compartments confer different cardiometabolic risk in children and adolescents. Mayo Clin Proc.

[25] Samouda H, De Beaufort C, Stranges S, Hirsch M, Van Nieuwenhuyse JP, Dooms G (2016). Cardiometabolic risk: leg fat is protective during childhood. Pediatr Diabetes.

[26] Weber D, Leonard M, Shults J, Zemel B (2014). A comparison of fat and lean body mass index to BMI for the identification of metabolic syndrome in children and adolescents. J Clin Endocrinol Metab.

[27] Malden D, Lacey B, Emberson J, Karpe F, Allen N, Bennett D (2019). Body fat distribution and systolic blood pressure in 10,000 adults with whole-body imaging: UK biobank and Oxford biobank. Obesity (Silver Spring).

[28] Zhang YX, Wang SR (2011). Relation of body mass index, fat mass index and fat-free mass index to blood pressure in children aged 7–12 in Shandong China. Ann Hum Biol.

[29] Ying-Xiu Z, Da-Yong S, Jing-Yang Z, Jin-Shan Z, Zun-Hua C (2014). Blood pressure among children and adolescents with normal weight but large waist circumference in Shandong China. Eur J Pediatr.

[30] Pazin DC, Rosaneli CF, Olandoski M, Oliveira ERN, Baena CP, Figueredo AS (2017). Waist circumference is associated with blood pressure in children with normal body mass index: a cross-sectional analysis of 3,417 school children. Arq Bras Cardiol.

[31] Ma J, Wang Z, Dong B, Song Y, Hu P, Zhang B (2012). Body fat and blood pressure: comparison of blood pressure measurements in Chinese children with different body fat levels. Br J Nutr.

[32] Julius S, Majahalme S, Nesbitt S, Grant E, Kaciroti N, Ombao H, Vriz O (2002). A "gender blind" relationship of lean body mass and blood pressure in the Tecumseh study. Am J Hypertens.

[33] Sidoti A, Nigrelli S, Rosati A, Bigazzi R, Caprioli R, Fanelli R (2017). Body mass index, fat free mass, uric acid, and renal function as blood pressure levels determinants in young adults. Nephrology (Carlton).

[34] Xu R, Zhang X, Zhou Y, Wan Y, Gao X (2019). Percentage of free fat mass is associated with elevated blood pressure in healthy Chinese children. Hypertens Res.

[35] Wells JC (2007). Sexual dimorphism of body composition. Best Pract Res Clin Endocrinol Metab.

[36] Mathieu P, Poirier P, Pibarot P, Lemieux I, Després J (2009). Visceral obesity: the link among inflammation, hypertension, and cardiovascular disease. Hypertension.

[37] Ritchie S, Connell J (2007). The link between abdominal obesity, metabolic syndrome and cardiovascular disease. NMCD.

[38] Jonas M, Kurylowicz A, Bartoszewicz Z, Lisik W, Jonas M, Wierzbicki Z (2015). Interleukins 6 and 15 levels are higher in subcutaneous adipose tissue, but obesity is associated with their increased content in visceral fat depots. Int J Mol Sci.

[39] Subasinghe A, Wark J, Gorelik A, Callegari E, Garland S (2017). The association between inflammation, obesity and elevated blood pressure in 16–25-year-old females. J Hum Hypertens.

[40] Chow W, Cheung B, Tso A, Xu A, Wat N, Fong C (2007). Hypoadiponectinemia as a predictor for the development of hypertension: a 5-year prospective study. Hypertension.

